# Effect of Addition of Polyurea as an Aggregate in Mortars: Analysis of Microstructure and Strength

**DOI:** 10.3390/polym14091753

**Published:** 2022-04-26

**Authors:** Hernan Chacon, Heidis Cano, Joaquin Hernández Fernández, Yoleima Guerra, Esneyder Puello-Polo, John Fredy Ríos-Rojas, Yolima Ruiz

**Affiliations:** 1 Department of Civil and Environmental Engineering, Universidad de la Costa, Calle 58#55-66, Barranquilla 080002, Atlántico, Colombia; hchacon2@cuc.edu.co (H.C.); yruiz4@cuc.edu.co (Y.R.); 2 Chemical Engineering Program, School of Engineering, Universidad Tecnológica de Bolívar, Parque Industrial y Tecnológico Carlos Vélez Pombo km 1 Vía, Turbaco 130001, Bolívar, Colombia; 3 Centro de Investigación en Ciencias e Ingenierías, Cecopat & A, Cartagena 131001, Bolívar, Colombia; stdpractice1@cecopat.com; 4 Grupo de Investigación en Oxi/Hidrotratamiento Catalítico y Nuevos Materiales, Programa de Química-Ciencias Básicas Universidad del Atlántico, Barranquilla 080003, Atlántico, Colombia; esneyderpuello@mail.uniatlantico.edu.co; 5 Department of Mechanical, Electronic and Biomedical Engineering, Antonio Nariño University, Bogotá 111321, Colombia; johnri@uan.edu.co

**Keywords:** mortar, polyurea, characterization, construction

## Abstract

The addition of polymers in construction is a new tendency and an important step toward the production of structures with better functional properties. This work investigates the addition of polyurea (PU) as a polymeric material in mortars. Polymer mortars were manufactured with the addition of polyurea retained in different sieves (T50 and T100) and different concentrations (2% and 5%). The characterization of the, polyurea (PU)control mortar (PU0%) and manufactured polyurea mortars (PU2%T50, PU5%T50, PU2%T100, and PU5%T100) was conducted by means of morphological analysis, SEM, XRF, TGA, and a compressive strength test of hydraulic mortars. The results show that mortars with polyurea retained in sieve 100 with a particle size of 150 μm exhibit better thermal behavior and a greater resistance to compression with a concentration of 5% polyurea with respect to the other samples. The present work reveals that polyurea retained in sieve 100 can be considered as a polymeric additive for mortars, indicating that it could be a candidate for applications such as construction.

## 1. Introduction

Currently, the construction industry presents a substantial challenge for material engineers considering the concept of sustainable development, which is a widely discussed issue. The use of polymers represents a new perspective, especially in the construction industry, as materials that provide functional properties.

Masonry consists of joining single units together with mortar. This type of construction is very popular and is widely used for small and medium residences, particularly in the construction of interior and external structural walls, the facades of buildings, and nonstructural divisions, which must withstand different loads and stresses making them prone to collapse [1,2].

In mountainous regions, for the opening of highway construction projects or highway expansion, the use of explosives is necessary. In this sense, some explosions indirectly affect the houses of local inhabitants, causing cracks and fractures in walls and, in some cases, in the structural systems. For this reason, in masonry, the materials selected for construction are important, especially the mortar, which is considered as the material joining two masonry units together. Furthermore, to improve the resistance of masonry walls, it is common to use mortar as a coating, confining the concrete elements of the wall. This system, however, has only a modest ability to dissipate energy in the inelastic range. In this sense, mortar must meet various requirements depending on its use, i.e., for joining masonry (glue mortar), as a filling mortar (grouting), or for coating structures (mortar or plaster for interior walls). However, the NSR 10 standard [3,4] establishes that the function of mortar is to adhere masonry units, with a dosage of 7.5 MPa (75kgf/cm^2^) considered appropriate to guarantee quality [3].

The minimum value of compression at 28 days varies depending on the materials added to potentiate the properties of mortars, including the addition of polymers to the traditional mixture of cement, sand, and water [4]. This addition improves the durability, workability, and mechanical properties [5].

Mortars have been studied with different additions of polymers, such as polyurethane, fiberglass, styrene–acrylate copolymers, and vinyl copolymers [6,7,8,9,10,11,12], obtaining results such as a reduction in compressive strength, as well as an improvement in performance with respect to adhesion, flexibility, permeability, etc. Therefore, these mortars can meet the present and future requirements in terms of construction and can face different stresses and loads.

There are several polymers suitable for addition to mortars that have not been studied. One example is polyurea (PU), which is a material that has been widely used in construction as a waterproof material to withstand different mechanical stresses and deformations [13]. PU is a synthetic polymer that is generated by the reaction of diisocyanate with diamine. The polymerization reaction is similar to that of polyurethane, with both undergoing condensation polymerization. However, the difference lies in the bond, with that of polyurea formed with an amine and that of polyurethane formed with a hydroxyl group [14]. Its properties include high flexibility, tensile strength, chemical resistance, high toughness when tearing, high chemical resistance, high durability, low flammability in elastomeric copolymers, high shear resistance, and generally improved thermal properties.

The addition of a granular material in the formation of the mortar must consider the size of the particles because previous studies have shown that failures that occur in mortars can occur more frequently at the weakest interaction in the zone interface between the cement and aggregate [15]. The concentration of tensile stress around the aggregate grains (the transit zone) between materials with different properties can cause concrete failure. For this reason, the bond between the cement mix and the aggregate is important. Thus, the surface of an aggregate that affects the transit zone is an important factor [15,16].

In the addition of a polymeric material to the mortar mixture, the size and surface area of the component will affect the compressive strength of the mortar; therefore, in this study, two particle sizes of PU and different concentrations were used to determine the optimum parameters for addition to mortars.

In this research, the addition of PU as a polymeric material in mortars is proposed. To determine the influence of PU on the mortars, the compressive strength of hydraulic mortars is investigated. Furthermore, the PU and the polyurea mortars fabricated are analyzed using granulometric analysis, thermogravimetric analysis (TGA), X-ray fluorescence (XRF), and SEM analysis.

## 2. Materials and Methods

A description of the materials and methods used in this research is presented below.

### 2.1. Materials

#### 2.1.1. Polyurea (PU)

**Figure 1:**
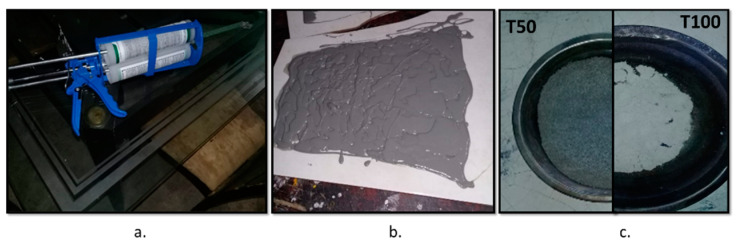
Preparation of PU: (**a**) EUCO QWIKJOINT 200 polyurea; (**b**) gray color of PU; (**c**) PU retained in sieve 50 and sieve 100.

The polymeric material used was EUCO QWIKJOINT 200 [17,18], which is a semirigid, fast-setting polyurea, mostly used in construction in concrete-type industrial floors and for the filling of control joints. The PU used is composed of two parts: part A is a transparent amber color and is composed of a mixture of two dissociations, and part B is a pigmented gray color and is composed of three components, as shown in Table 1. When mixed in a proportion of 1:1, a gray color similar to that of concrete is obtained (see Figure 1a,b).

The PU was prepared by mixing the two components, and it was allowed to dry for a period of 24 h. Later, it was stripped and sieved, thus obtaining various PU particle sizes, from which those retained in sieve 50 and sieve 100 (particle sizes of 300 and 150 μm, respectively) were extracted to be used for manufacturing the mortar (see Figure 1c).

#### 2.1.2. Sand, Cement, and Water

A well-graded clay sand (SW-SC) was used. The SW-SC was characterized under the ASTM D422-63 standard [19], and its granulometric profile is shown in Table 2. The sand presented a natural humidity of 5.4% and a fineness module of 2.1.

Ordinary Portland cement was used according to NTC 121 and ASTM C1157 regulations [20,21]. This cement is used for the construction of mortars in general, as well as concrete structures, such as beams, columns, slabs, walls, and foundations [22]. Tap water was used according to the Colombian regulations for drinking water.

#### 2.1.3. Mix Design and Polymer Mortar Manufacture

The combinations obtained with various quantities of sand, cement, water, and PU are shown in Table 3. The preparation of mortars was carried out according to the standard ASTM C109/C109M-21 [23]. Mortar cubes with different curing ages of 7, 14, and 28 days were prepared.

After curing, the mortar was removed from the lime water mixture and allowed to dry for different periods of time depending on curing age. Mortars cured for 7 days were left to dry for a period of ~3 h, those cured for 14 days were left to dry for a period of ~6 h, and those cured for 28 days were left to dry for a period of ~12 h.

### 2.2. Characterization

#### 2.2.1. X-ray Fluorescence

To determine the effect of PU on the formation of the main oxides in cement, a quantitative compositional analysis employing X-ray fluorescence (XRF) was performed using Thermo ARL Optim’X WDXRF equipment. The analysis was conducted on samples cured for 28 days. Minerals are responsible for the characteristics and properties of cement, such as the initial strength provided by tricalcium silicate (3CaO·SiO_2_ or alite) or the strength granted by concrete over time provided by dicalcium silicate (2CaO·SiO_2_ or belite). Tricalcium aluminate (Al_2_O_3_·3CaO or celite) is essential for the chemical reactions of concrete to occur, whereas tetracalcium aluminoferrite (Al_2_O_3_·4CaO·Fe_2_O_3_ or ferrite) is necessary for slow hardening [24,25].

#### 2.2.2. Particle Size

A particle size analysis was conducted to determine the characteristic size profile of the different polyurea mortar samples, thereby enabling the relationship between particle behavior and polyurea mortar properties to be established. For this, 5 mg of polyurea mortar was taken and analyzed using Microtrac equipment, which quantified the number of particles present in the sample and characterized its morphological parameters, such as size, area surface, volume, and diameter.

#### 2.2.3. Scanning Electron Microscopy (SEM)

Microstructural analysis of the PU and polyurea mortars was performed using scanning electron microscopy (SEM), employing a Thermoionic SEM JEOL JSM-6490LV. A 2 × 2 cm sample of polyurea mortars was used. The sample was pretreated with dehydration followed by metallization. This analysis was performed to observe the interfacial adhesions among the added polymer, cement, and aggregates, as well as the hydration products formed. Furthermore, a semiquantitative analysis of the elemental composition was performed using energy-dispersive spectroscopy (EDS) coupled to SEM.

#### 2.2.4. Thermogravimetric Analysis (TGA)

TGA was performed to determine the thermal stability of pure PU with different particle sizes and of polyurea mortars formed with the addition of PU at different concentrations. The instrument used for this test was a Perkin Elmer TGA7. In a typical experiment, a sample weighing between 10 and 20 mg is uniformly placed in an alumina crucible and pyrolyzed under a flow of N_2_, heating the samples from 50 to 850 °C [9,26,27].

#### 2.2.5. Compressive Strength Test

A compressive strength test was performed using equipment in 30000100 mode, applying a relative load ratio in the range of 900–1800 N/s according to the standard ASTM C109/C109M-21 [23]. First, cubes of 50 mm were fabricated and then dried for 1 day in a mold, before being immersed in a mixture of lime water until the tests were carried out after 7, 14, and 28 days of curing [14].

## 3. Results and Discussion

### 3.1. Microstructural Characterization of Polymer Mortars

#### 3.1.1. X-ray Fluorescence

Table 4 shows the results of the XRF analysis. The percentages of oxides found in the polyurea mortars (PU2%T100, PU5%T100, PU2%T50, and PU5%T50) and in the control mortar (PU0%) are presented.

**Figure 2:**
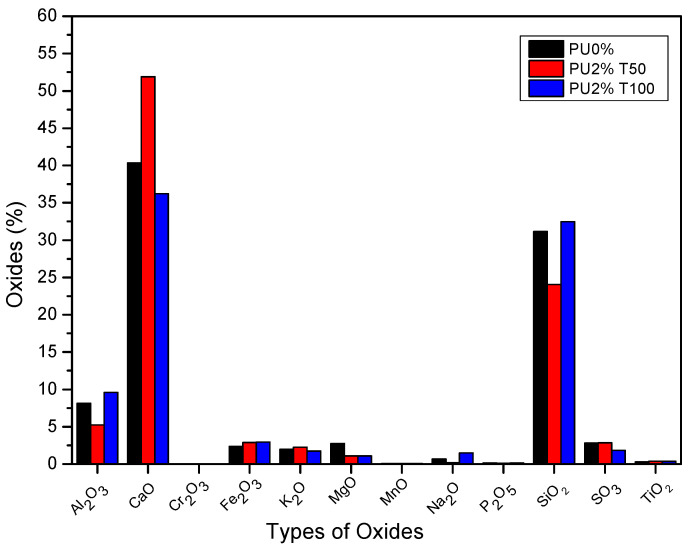
Percentages of different minerals in the cement of polyurea mortars with 2% addition of PU and the use of different sieves (T50 and T100).

Figure 2 shows the percentages of different minerals in the cement of the polyurea mortars with 2% addition of PU and the use of different sieves (T50 and T100). It can be observed that the PU2%T100 mortar presented different percentages of oxides with respect to the control mortar (PU0%), especially in terms of calcium oxide (CaO), with values 2.65%–4% lower than those of PU0%. Furthermore, it can be observed that the PU2%T50 polyurea mortar presented marked variations compared to PU0%, with an 11.5% increase in the concentration of CaO, a 2.9% decrease in the concentration of aluminum oxide (Al_2_O_3_), and a 7.1% decrease in the concentration of silicon oxide (SiO_2_) compared to PU0%. According to the results, the formation of calcium, aluminates, and silicates in the mortars was influenced by the addition of polyurea, especially for PU2%T50.

**Figure 3:**
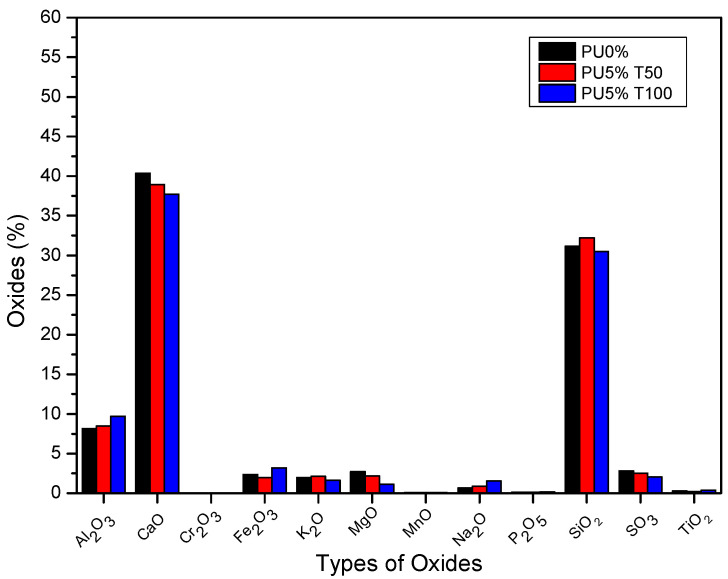
Percentages of different minerals in the cement of polyurea mortars with 5% addition of PU and the use of different sieves (T50 and T100).

Figure 3 shows the percentages of different minerals in the cement of the polyurea mortars with 5% addition of PU and the use of different sieves (T50 and T100). In general, PU5%T50 and PU5%T100 polyurea mortars maintained similar values to those of PU0%, with small variations in CaO, Al_2_O_3_, and SiO_2_. The formation of calcium, aluminates, and silicates in the mortars was not influenced by the addition of polyurea.

It is known that the presence of these oxides affects the production of dicalcium silicate (2CaO·SiO_2_ or belite) and tricalcium silicate (3CaO·SiO_2_ or alite), both responsible for the strength of the concrete. The former is related to the acquisition of strength over time, while the latter is related to the initial strength. Therefore, it was expected that the resistance of the PU2%T50 mortar would differ significantly with respect to the other mortars. However, all mortars generally had normal values within the ranges referenced in the literature for aluminum, calcium, and silicon oxide [7,28,29,30].

The variation in the percentages of oxides present in the samples can be affected by the components of the aggregate used for the manufacture of the mortars. When using well-graded sand, the particle size distribution of the aggregate is guaranteed. Although not all samples presented the same amounts of metal compounds in the aggregate, the values obtained were within acceptable ranges [31,32,33].

#### 3.1.2. Particle Size

**Figure 4:**
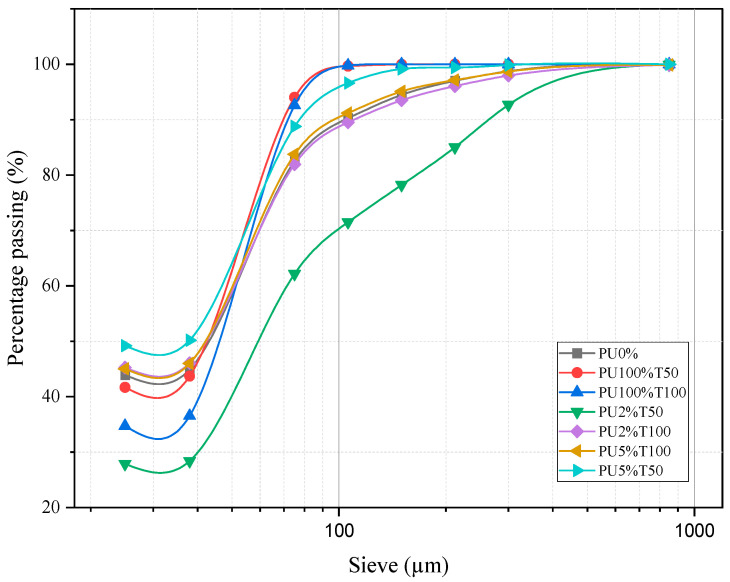
Granulometry profile of PU and polyurea mortar samples.

Figure 4 shows the granulometric profile of the polyurea mortars analyzed. Varying behaviors were observed among the mortars. PU2%T50 can be highlighted as having a granulometric profile below that of PU0%. Likewise, it can be observed that the samples PU2%T100 and PU5%T100 had similar profiles to that of PU0%. Figure 4 also shows the granulometric profile of pure polyurea with different sieve sizes (PU100%T50 and PU100%T100).

The samples with a similar profile to that of PU0% were those using polyurea retained in sieve 100. This is because the smaller particles were conducive to the granulometry not being affected. In contrast, the samples using polyurea retained in sieve 50 presented different behaviors according to the concentration, obtaining curves above and below that of the control mortar when the concentrations were higher and lower, respectively.

**Figure 5:**
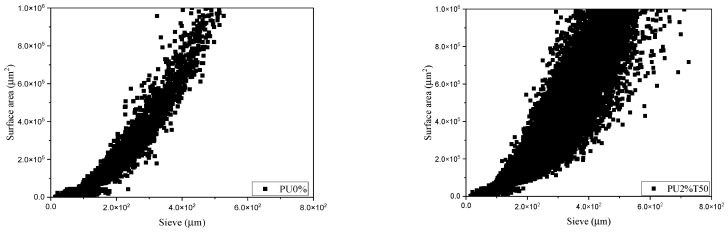

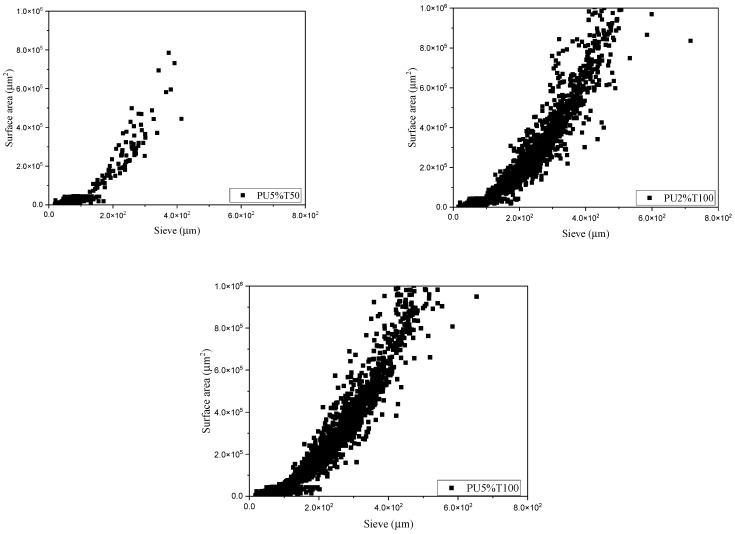
Dispersion analysis: size vs. surface area of polyurea mortars.

It is well known that the surface area of particles allows interactions between components. Figure 5 reveals that the samples presenting a greater dispersion of the particles presented a lower resistance with a certain linearity. In the case of samples retained in sieve 50, a greater dispersion can be observed in the PU2%T50 sample with respect to the PU5%T50 sample, a behavior that was not replicated in the PU2%T100 and PU5%T100 samples.

#### 3.1.3. SEM Analysis

**Figure 6:**
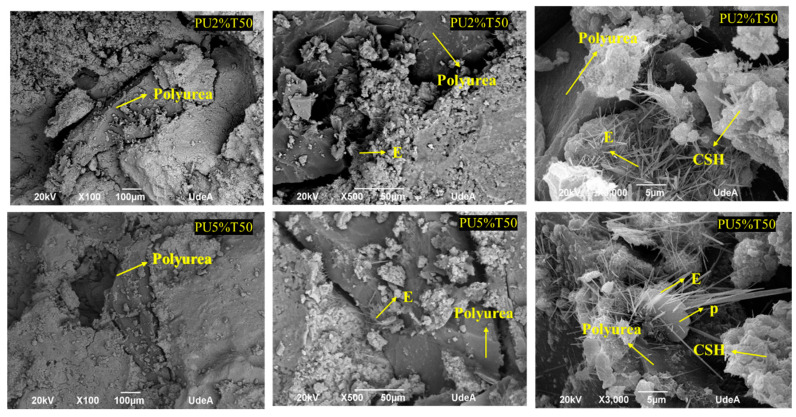
SEM analysis of mortars with polyurea retained in sieve 50 (PU2%T50 and PU5%T50).

**Figure 7:**
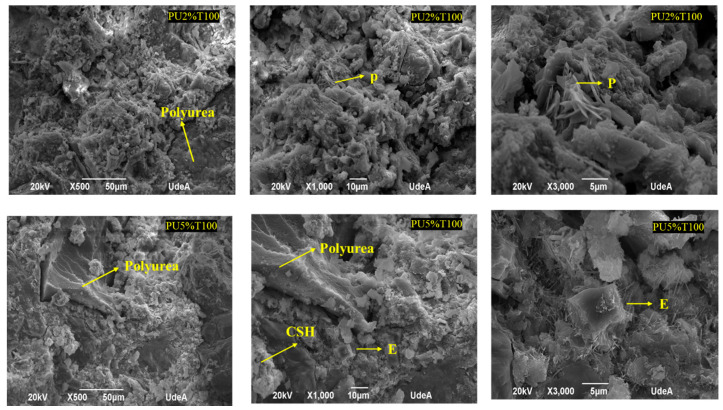
SEM analysis of mortars with polyurea retained in sieve 100 (PU2%T100 and PU5%T100).

Figure 6 and Figure 7 show the microstructure of the polyurea mortars manufactured with different additions of polyurea. It can be observed from the microphotographs that PU was present in all mortars, in addition to cement hydrates, such as thin needles of hydrated calcium sulfoaluminate (E; ettringite), hydrated calcium silicate crystals (CSHs), and portlandite (P) forming boards or chips within a gap-free space.

**Figure 8:**
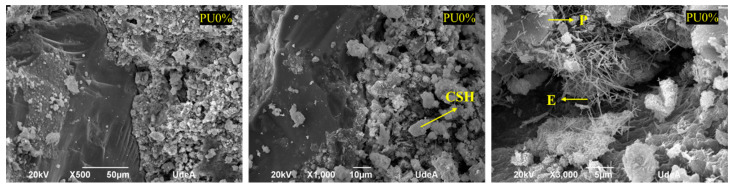
SEM analysis of mortar control PU0%.

The SEM analysis revealed an interaction between the different components of the mortar and the presence of spaces or small interstices between the PU and the mortar mixture. Thus, the adhesion between the two components was incomplete. These spaces were more evident in the samples of PU retained in sieve 50 due to the larger particles not promoting adhesion between the PU and the mortar mixture. The presence of these interstices makes the material more susceptible to ruptures, thus leading to lower resistance. This has also been observed by other authors [34,35], who used various polymeric materials in mortars, determining that the tensile stress around aggregate grains between materials with different properties can generate concrete failure. However, regardless of the percentage of PU addition, it was possible to locate cement hydrates attached to PU. In general, the hydration products formed were similar for all mortar samples, including the control PU0% (see Figure 8). Fine needles of ettringite (E), portlandite plates (P), and hydrated calcium silicate amorphous phase (C-S-H) were observed.

**Figure 9:**
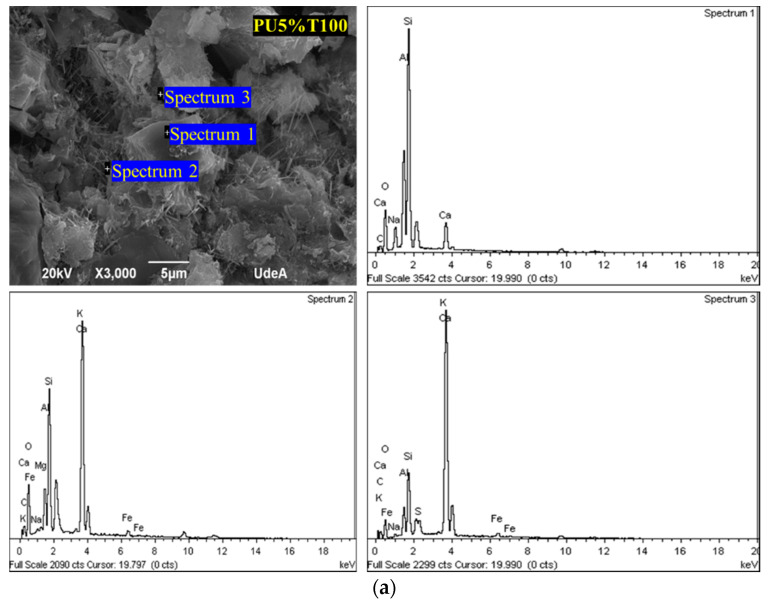

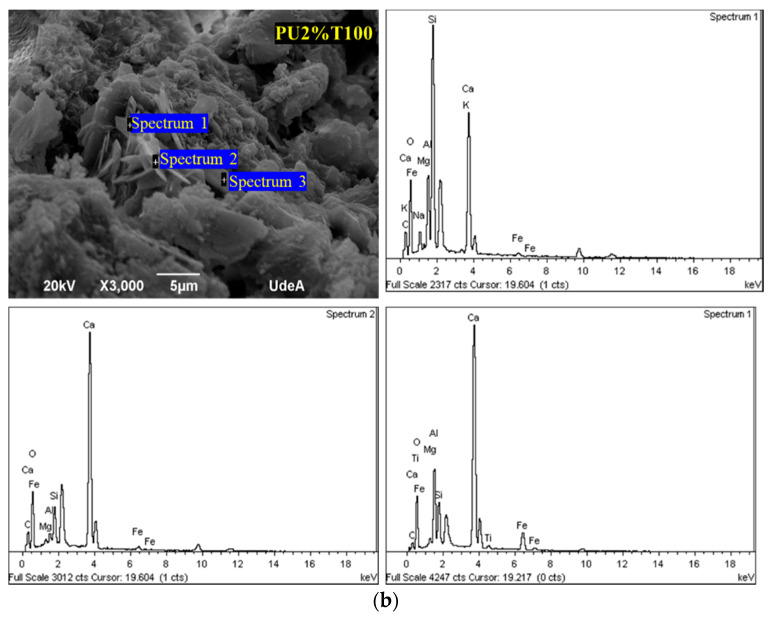
SEM–EDS analysis of mortars with polyurea retained in sieve 100: (**a**) PU5%T100; (**b**) PU2%T100.

Figure 9 shows the EDS compositional analysis of the polymer mortars PU5%T100 and PU2%T100. The regions of the spectra allowing the identification of components are highlighted. This allowed the structures to be differentiated according to the components present in the hydrated products of the cement, suggesting that the addition of PU did not affect the normal formation of the mortar.

#### 3.1.4. TGA

**Figure 10:**
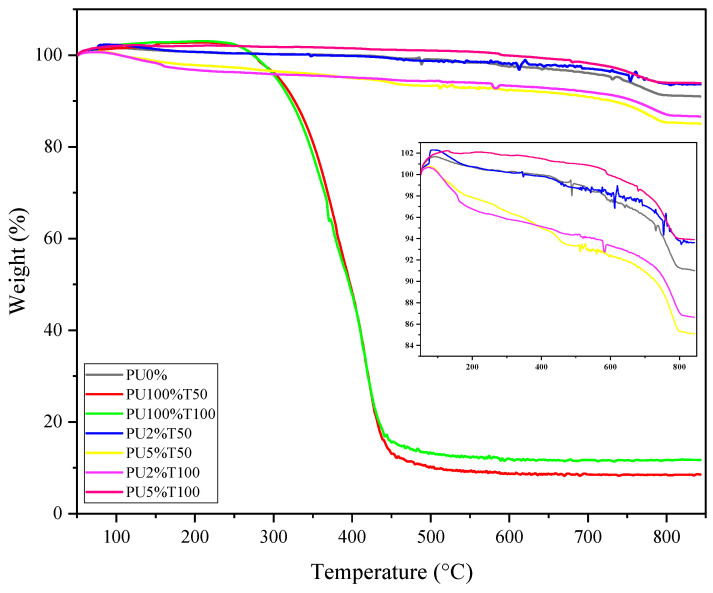
TGA of the samples of PU, polyurea mortars, and control mortar (PU0%).

Figure 10 shows the thermogravimetric analysis of pure polyurea (PU100%T50 and PU100%T100), polyurea mortars (PU2%T50, PU5%T50, PU2%T100, and PU5%T100), and the control mortar (PU0%). The TGA of the polyurea mortar samples and the pure polyurea showed that the typical behavior of polyurea was not replicated by the mortar samples; however, it could be determined that the addition of PU decreased the percentage weight loss with temperature as a function of particle size and concentration. The sample with a smaller particle size and a higher concentration (sample PU5%T100) presented superior behavior in terms of weight maintenance compared to the sample with a larger particle size and a lower concentration (sample PU2%T50).

The decomposition stages of mortars can be differentiated according to the temperature range. The first window of weight loss between 20 and 110 °C corresponded to the evaporation of free water. The second window of weight loss between 120 and 400 °C corresponded to the loss of water from the hydrated calcium silicate (CSH) gel and the minority phases of aluminates and sulfoaluminates. The third window of weight loss between 410 and 530 °C corresponded to the decomposition of calcium hydroxide, whereby water was released and calcium oxide remained. The final window of weight loss between 550 and 950 °C corresponded to the decomposition of the calcium carbonate present in the cementitious material [36]. In the case of pure polyurea, the degradation of urea bonds was identified. However, when analyzing the mixture of mortars with polyurea, the effect of polyurea on mortar decomposition was mediated by two factors, the concentration and the size of the polyurea, with the particle size being more influential due to it improving the interaction between components. The variations between different mortars may have been due to the number of degraded urea-type bonds, the dispersion of particles in the mortar, and their interaction with the components of the mortar. As fewer interstices were identified by SEM analysis in the mortar with polyurea retained in sieve 100, the effect of the concentration was more noticeable than for the mortar with polyurea retained in sieve 50. Overall, it can be observed that adding PU in different concentrations and sizes affected the thermal stability of the mortar, which translated into variations in the amount of material lost at each stage.

However, Figure 10 shows an increase in weight in the TGA thermograms for all samples. It is probable that the formation and breaking of bubbles inside the material caused changes in pressure, which can cause alterations in the test, and generated a positive oscillation in the thermobalance. This means that the increase is due to the positive oscillation of the thermobalance, which is a result of the gases generated or due to the bursting of bubbles. This might be called positive instrumental noise.

The initial decomposition temperature (T_initial_), the 50 wt.% loss temperature (T_50%_), and the final decomposition temperature (T_end_) are shown in Table 5. Significant variations can be observed in T_50%_ for the PU5%T50 and PU2%T100 samples, where the temperature dropped by about 20% with respect to the control sample.

In some cases, the outermost layer of a polymer does not allow gases to migrate to the outside because, during melting or relaunching, it becomes more impermeable. This results in the formation of bubbles inside the material, which increases the pressure until the sample is ruptured, reflected as a sudden loss of mass. It is important to note that this can occur at different temperatures [37,38].

TGA allows the thermal behavior of mortars to be determined so that the influence of the polymer composition on the thermal degradation can also be determined. In the case of PU, its applicability depends on its particle size and concentration due to their influence on the thermal stability and resistance of the mortars.

#### 3.1.5. Resistance

Table 6 shows the results of the compression test of PU0% and the mortar samples retained in sieves 50 and 100 with different concentrations of PU (PU2%T50, PU5%T50, PU2%T100, and PU5%T100) after 7, 14, and 28 days of curing. The resistance specified was 20.68 MPa for all tests. In general, a lower resistance was presented with respect to the control sample after 28 days of curing. In the case of mortars with PU retained in sieve 100, a greater resistance with respect to the control sample was observed after 28 days of curing for both concentrations, with better results recorded for the sample with 2% PU, as shown in Table 6. From the data obtained, we can deduce that the mortars with different concentrations of PU retained in sieve 50 had a lower resistance than PU0%, whereas the mortars with PU retained in sieve 100 exhibited increased resistance after 28 days of curing.

It can be highlighted that the mortars with PU retained in sieve 100 (PU2%T100 and PU5%T100) were inferior to the remaining samples after 7 and 14 days of curing. This could be due to the presence of water in the pores of the samples, which hindered the development of bonds between the mortar components and the polyurea, thus affecting the resistance of the material.

In general, it can be established that adding PU with a particle size of 150 μm (sieve 100) promoted an increase in compressive strength after 28 days of curing for both concentrations (PU2%T100 and PU5%T100). The smaller dimension of PU particles enables better penetration of the pores produced in the mortar, allowing interactions between the PU and the hydrated mortar. This is due to the influence of the aggregate surface area on the formation of ruptures in the mortars. A smaller particle size and surface area result in better compression of mortars [39,40].

## 4. Conclusions

According to the results of this experimental study, the following conclusions can be drawn:

- The addition of polyurea affected the sample profile when the particle size was large.
- SEM analysis revealed the presence of hydrates typical of cement.
- The thermal stability of the mortars was influenced by the addition of polyurea, as determined by TGA. The sample with a smaller particle size and higher concentration of polyurea (PU5%T100) presented better results with respect to conventional mortar in terms of weight maintenance.
- After 28 days of curing, polyurea with a small particle size improved the compressive strength of the sample.
- Taken together, these findings suggest that polyurea retained in sieve 100 can be considered as a polymeric additive for mortars, indicating that it could be a candidate for applications such as the construction of interior and external structural walls, building facades, and nonstructural divisions, where durability, workability, and mechanical properties are required.

## Figures and Tables

**Table 1:** Chemical composition of the PU used [18].

Chemical Identity	CAS	Concentration in Percentage (%*w*/*w*)	
		Part A	Part B
4,4′-Methylene bis(phenylisocyanate)	101-68-8	15–40%	-
Diphenylmethane diisocyanate	26447-40-5	10–30%	-
Diethyltoluenediamine	68479-98-1	-	15–40%
Propoxylated Amine	102-60-3	-	3–7%
Amorphous silica	7631-86-9	-	1–5%
Titanium dioxide	13463-67-7	-	0.5–1.5%

**Table 2:** Granulometry of clay sand.

Sieves	Opening (mm)	Retained Mass (g)	% Retained	Cumulative Retained %	% PASS
No.					
1″	25.000				
3/4″	19.000				
1/2″	12.500				
3/8″	9.500	0.0	0.0	0.0	100.0
4	4.750	1.9	0.0	0.0	100.0
8	2.360	13.8	0.3	0.3	99.7
16	1.180	121.5	2.5	2.9	97.1
30	0.600	1.2058	25.1	28.0	72.0
50	0.300	2.4758	51.6	79.5	20.5
100	0.150	839.2	17.5	97.0	3.0
200	0.075	31.1	0.65	97.67	2.33
P 200	0.000	111.7	2.33	100.00	0.00
Starting Weight (g):		4800.8	Final Weight (g):		4689.1

**Table 3:** Mix design used for polymer mortar preparation.

Polymer Mortar	Number of Mortars	PU Addition (%)	PU Particle Size (μm) (Sieve)	Mix Design			
				Cement (g)	Sand (g)	Water (g)	PU (g)
PU0%	9	0	-	750	1736.4	390	-
PU2%T50	9	2	300 (50)	750	1736.4	390	14.25
PU5%T50	9	5	300 (50)	750	1736.4	390	37.5
PU2%T100	9	2	150 (100)	750	1736.4	390	14.25
PU5%T100	9	5	150 (100)	750	1736.4	390	37.5

**Table 4:** Quantification of oxides present in the samples determined by X-ray fluorescence.

Oxides	PU0%	PU2%T100	PU5%T100	PU2%T50	PU5%T50
	% p/p				
Al_2_O_3_	8.16	9.61	9.71	5.24	8.46
CaO	40.35	36.23	37.7	51.9	38.92
Cr_2_O_3_	0.022	0.009	0.008	0.023	0.01
Fe_2_O_3_	2.34	2.95	3.202	2.87	1.98
K_2_O	1.98	1.75	1.62	2.25	2.11
MgO	2.73	1.08	1.16	1.08	2.2
MnO	0.054	0.055	0.059	0.071	0.053
Na_2_O	0.675	1.49	1.55	0.16	0.829
P_2_O_5_	0.126	0.154	0.163	0.094	0.143
SiO_2_	31.17	32.48	30.49	24.05	32.21
SO_3_	2.81	1.84	2.04	2.83	2.52
TiO_2_	0.274	0.331	0.397	0.381	0.217
^1^ LOI 550 °C	3.11	4.686	4.95	3.225	4.012
LOI 950 °C	9.046	11.763	11.643	8.73	10.149

^1^ LOI: loss on ignition at the reference temperature.

**Table 5:** Decomposition temperatures of samples.

Samples	Decomposition Temperature °C		
	T_inicial_	T50%	T_end_
PU0%	50.8	722.55	842.63
PU100%T50	51.2	391.28	843.05
PU100%T100	51.2	386.17	843.15
PU2%T50	51.3	715.47	833.7
PU5%T50	51.1	573.43	841.59
PU2%T100	51.1	579.33	841.11
PU5%T100	51.1	740.82	839.38

**Table 6:** Resistance test for control sample (PU0%) and resistance of mortars with polyurea retained in sieves 50 and 100.

	Age (Days)	Maximum Load (kN)	Average Stress (Mpa)	Standard Deviation (σ)	Average Resistance (%)
PU0%	7	36.03	14.41	0.18	70
	14	44.53	17.81	0.08	86
	28	55.37	22.14	0.23	107
PU2%T50	7	24.03	9.61	0.13	46.70
	14	49.43	19.77	0.10	95.30
	28	38.00	15.20	0.11	73.30
PU5%T50	7	28.93	11.57	0.05	56.00
	14	38.36	15.34	0.08	74.30
	28	33.33	13.33	0.19	64.30
PU2%T10	7	29,30	11.72	0.31	57
	14	37.43	14.97	0.26	72.3
	28	62.20	24.88	0.06	120.3
PU5%T100	7	24.13	9.65	0.18	47
	14	38.57	15.43	0.15	74.7
	28	60.73	24.29	0.15	117.3
